# Natural course of coronary artery calcium progression in Asian population with an initial score of zero

**DOI:** 10.1186/s12872-020-01498-x

**Published:** 2020-05-06

**Authors:** Yi-Wen Shen, Yun-Ju Wu, Yi-Chi Hung, Chia-Chi Hsiao, Shan-Ho Chan, Guang-Yuan Mar, Ming-Ting Wu, Fu-Zong Wu

**Affiliations:** 1 Department of Radiology, Kaohsiung Veterans General Hospital, Kaohsiung, Kaohsiung, Taiwan; 2 Department of Medical Imaging and Radiological Sciences, Kaohsiung Medical University, Kaohsiung, Taiwan; 3 Department of Medical Imaging and Radiology, Shu-Zen Junior College of Medicine and Management, Kaohsiung, Taiwan; 4 Physical Examination Center, Kaohsiung Veterans General Hospital, Kaohsiung, Taiwan; 5 Faculty of Medicine, School of Medicine, National Yang-Ming University, Taipei, Taiwan

**Keywords:** Coronary artery calcium, Zero score, vulnerable plaque

## Abstract

**Background:**

We aimed to investigate the natural course of coronary artery calcium progression in an Asian population with a baseline coronary artery calcium (CAC) score of zero, and to determine subclinical coronary atherosclerosis.

**Methods:**

Four hundred fifty-nine subjects with at least two CAC scans with an initial score of zero were included. CAC progression (+) was defined by the development of any CAC (i.e., CAC > 0) during subsequent CT scans. Clinical characteristics and Framingham risk profiles were also recorded.

**Results:**

Among 459 subjects, 106 (23.09%) experienced CAC progression during the average follow-up period of 5.71 ± 2.68 years. Older age, male gender, HDL-C, total cholesterol and higher Framingham risk score were independently associated with CAC progression. Framingham risk score had the better discriminative ability (AUC = 0.660) to predict CAC progression compared to the other parameters with a sensitivity of 75.24% and specificity of 53.95%. For the double zero score with coronary artery atherosclerosis prediction, older age, triglycerides, hypertension, and Framingham risk score were significantly associated with these events. Among these parameters, Framingham risk score may be a relatively acceptable parameter with high negative predictive (NPV = 96.4%) value to rule out double zero score with obstructive coronary artery atherosclerosis scenario with an optimum cut-off value of <16.9 (AUC =0.652, sensitivity of 57.69%; specificity of 68.82%).

**Conclusions:**

A baseline zero CAC score in asymptomatic Chinese population with low to intermediate risk have a low incidence for CAC progression within the 5-years period. For CAC progression prediction, Framingham risk score with the cutoff < 11.1 may help confirm subjects at low risk to improve cardiovascular risk stratification and reclassification in the field of preventive cardiology.

## Background

Coronary artery calcification is considered being characteristic of subclinical atherosclerosis burden. In recent years, coronary artery calcium (CAC) scoring assessed by computed tomography has been proposed as a gatekeeper for non-invasive coronary artery diseases stratification and reclassification [1–7]. Many studies have investigated that CAC progression may be more predictive of future cardiac events than traditional cardiovascular risks [8–10]. Therefore CAC scanning has now been given a class IIa recommendation by the 2019 ACC/AHA Guideline on the Primary Prevention of Cardiovascular Disease for the use of CAC quantification in intermediate risk population to improve cardiovascular risk assessment [11–13].

Recent studies have demonstrated that individuals with calcium score of zero have a very low risk of coronary artery disease (CAD) with a warranty period of 5 years in Western population [9, 14–16]. There is limited evidence that absence of CAC has a protective effect against CAD in non-Western populations [8, 17]. The natural course of CAC progression in Asian population remains to be established according to different Framingham risk scores. In addition, recent studies have showed that absence of coronary artery calcification does not completely exclude obstructive coronary artery disease, but the prevalence is variable between 1 to 20% [18–23]. It is important to determine clinical parameters for predict obstructive CAD in this clinical scenario. Therefore, using an Asian cohort of low to intermediate risk stratification by Framingham Risk Score, we aimed to investigate the natural course of coronary artery calcium progression in an Asian population with a baseline CAC score of zero, and to determine CAC progression according to different risk stratification algorithm. In addition, we identified independent clinical parameters in prediction of CAC progression and obstructive coronary artery atherosclerosis in the clinical scenario of double-zero score.

## Methods

### Study population

Between April 2005 and March 2017, we identified 459 subjects with the baseline CAC score of zero who underwent 2 consecutive scans (CAC scan and coronary CT angiography) over a period of average 4.67 ± 2.46 years. Clinical characteristics and Framingham risk profiles were collected retrospectively by means of detail questionnaires such as age, sex, body mass index (BMI), hypertension, diabetes, current smoking, pack-year, total cholesterol, high-density lipoprotein cholesterol (HDL-C), low*-*density lipoprotein cholesterol (LDL-C), triglyceride, hemoglobin A1c (HbA1c) and Framingham risk score at the baseline health checkup. The institutional review board committees of our hospital approved this retrospective study and waived the need for informed consent.

### CAC scan

All CAC scans were performed with a 64-slice computed tomography (CT) (Aquilion 64; Toshiba Medical Systems), and 256-slice CT (Revolution CT, GE Healthcare, Milwaukee, USA). Coronary artery calcification was defined based on the Agatston method, for the quantification of the CAC score. In the Agatston method, calcification is defined as a hyper-attenuating lesion with a density > 130 Hounsfield units (HU) and area > 3 adjacent pixels (≥ 1 mm^3^) [24].

### Coronary CT angiography image acquisition and analysis

**Fig. 1:**
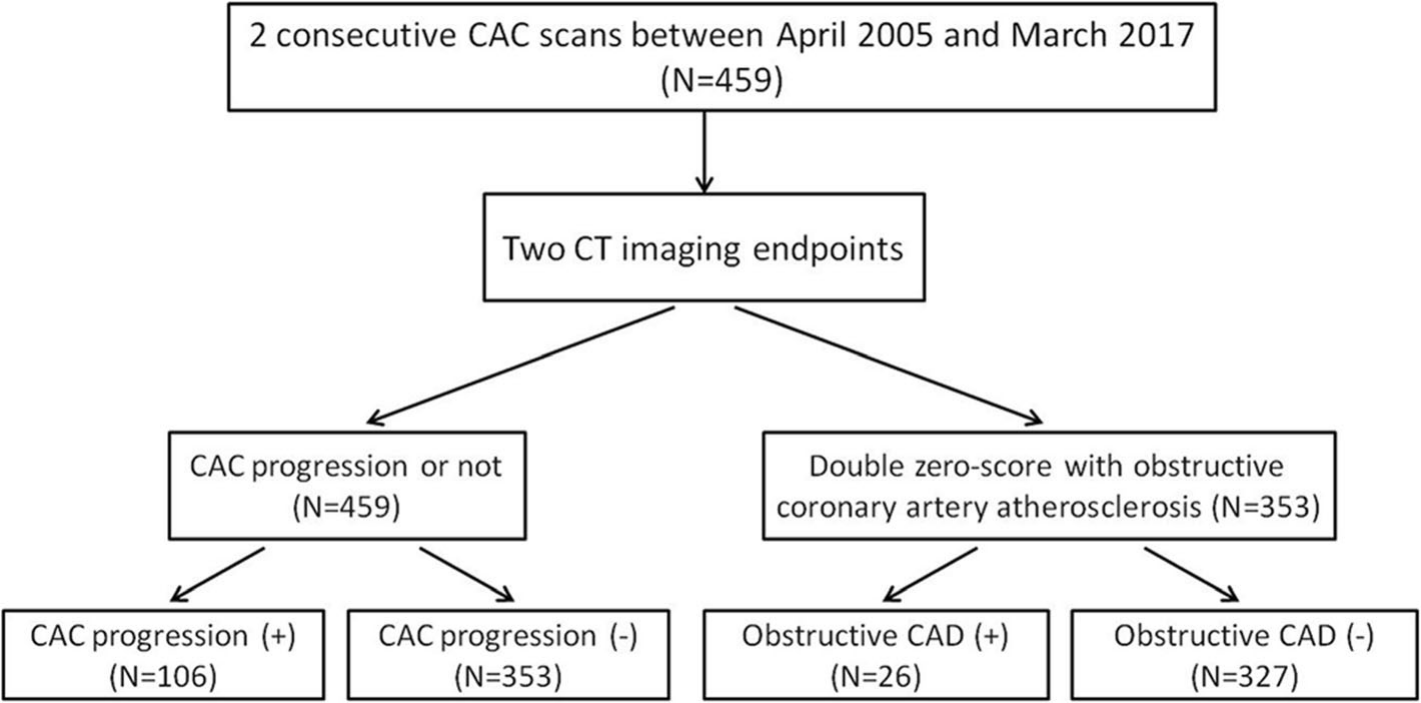
Flow chart showing the study population with baseline zero CAC score to determine subclinical coronary artery atherosclerosis in term of CAC progression and double zero score with obstructive coronary artery atherosclerosis

We performed ECG-gated CT angiography of the coronary arteries according to the guidelines of the society of cardiovascular computed tomography (SCCT) [25]. All patients were scanned with a 64-slice CT (Aquilion 64; Toshiba Medical Systems), and 256-slice CT (Revolution CT, GE Healthcare, Milwaukee, USA). We administered oral beta-blockers (metoprolol) if heart rate exceeded 65 beats per minute 1 h before the coronary CT angiography examination. All patients received 0.8 mg of sublingual nitroglycerin shortly prior to the contrast-enhanced scan. All coronary CT angiography images were acquired using prospective electrocardiogram (ECG) triggering. Images were acquired and reconstructed at diastole (75–81% of the R-R interval) or at systole (37–43% of the R-R interval) if heart rate was still above 70 bpm despite premedication. Two primary CT imaging endpoints were investigated using Cox regression analysis. Among the 459 participants with the baseline CAC score of zero, CAC progression (+) was defined by the development of any CAC (i.e., CAC > 0) during subsequent CT scans. In addition, we also evaluate the clinical scenario of double zero score with obstructive coronary artery atherosclerosis (defined as CAD-RADS≧3 or vulnerable plaques formation in the final round of the CT exam). Vulnerable plaques were defined as the presence of at least 2 features of spotty calcification, low attenuation plaque, or positive remodeling in final CT scans according to the SCCT guideline [26]. The study cohort was summarized according to the status of CAC progression and double zero score with obstructive coronary artery atherosclerosis shown in Fig. 1.

### Statistical analysis

All statistical analyses were performed using SPSS 22.0 for Windows (SPSS Inc., Chicago, IL). Continuous variables are presented as mean ± standard deviation (SD), and categorical variables as counts with proportions. Continuous variables were compared between groups using the Student’s t-test for normally distributed data and the Wilcoxon rank-sum test for non-normally distributed data. Categorical variables were compared between groups using the χ2 test or Fisher’s exact test, as appropriate.

The Kaplan-Meier curves were also used to estimate the distribution of the time to CAC progression according to the cardiovascular risk parameters, and differences among groups were evaluated with the log-rank test. Cox regression model was used to explore the impact of cardiovascular risk parameters on two CT imaging endpoints (CAC progression and double zero score with obstructive coronary artery atherosclerosis). A stepwise multivariate analysis was used to estimate the hazard ratios (HRs) based on the Cox regression model. The all Cox regression models retained a significant model (Omnibus test of model coefficients: *p* < 0.05). Collinearity among the included variables was tested using variance inflation factors (VIFs), and values less than 4.0 were considered to indicating non-collinearity. For CAC progression prediction, ROC curve analysis was used to determine the optimal cutoff value of cardiovascular risk parameters. For double zero score with obstructive coronary artery atherosclerosis, ROC curve analysis was used to determine the optimal cutoff value of cardiovascular risk parameters. Sensitivity, specificity, positive likelihood ratio (positive LR), negative likelihood ratio (negative LR), positive predictive value (PPV), negative predictive value (NPV) and diagnostic accuracy were determined from the optimal threshold by the Youden index.

## Result

The clinical characteristics of the study cohort with baseline zero CAC score are summarized in Table 1. The study cohort was comprised of 459 participants (mean age 51.42 ± 8.44), who had underwent 2 CT scans with an average of 4.67 ± 2.46 year. Of the 459 included participants with a baseline CAC score of zero, CAC progression was observed in 106 participants during the follow up, and the average time to CAC progression was 5.71 ± 2.68 years. There were no significant differences in diabetes, total cholesterol, LDL-C, triglyceride, and HbA1c among these two groups. Compared with the CAC progression (−) group, there was significantly higher age, male sex, BMI, hypertension, current smoking, pack-year, follow-up period, CAC scan in the final round, and Framingham risk score in the CAC progression (+) group. CAC progression (−) group had a higher proportion of HDL-C level than CAC progression (+) group.

**Table 1:** Baseline characteristics of 459 subjects with a baseline CAC score of zero and stratified according to CAC progression

	Total population (*n* = 459)	CAC progression (+) (*n* = 106)	CAC progression (−) (*n* = 353)	*P*-value
Age	51.42 ± 8.44	53.07 ± 8.06	50.92 ± 8.49	0.021
Gender (%, male)	311 (67.8%)	88 (83.0%)	223 (63.2%)	0.0001
BMI (kg/m2)	24.94 ± 3.46	25.73 ± 2.94	24.70 ± 3.57	0.007
Hypertension (%)	163 (35.7%)	51 (48.6%)	112 (31.9%)	0.002
Diabetes (%)	70 (15.3%)	20 (18.9%)	50 (14.2%)	0.281
Current smoking (%)	149 (34.0%)	44 (43.1%)	105 (31.3%)	0.032
Pack-year	9.89 ± 17.24	13.73 ± 20.06	8.72 ± 16.14	0.010
Total cholesterol (mg/dL)	207.56 ± 36.55	211.29 ± 38.64	206.44 ± 35.89	0.231
HDL-C (mg/dL)	46.46.43	42.93 ± 10.17	47.52 ± 14.11	0.002
LDL-C (mg/dL)	114.13 ± 28.63	117.20 ± 31.45	113.20 ± 27.71	0.208
Triglyceride (mg/dL)	157.03 ± 106.19	168.50 ± 96.38	153.58 ± 108.85	0.205
HbA1c (%)	5.87 ± 0.86	5.96 ± 0.81	5.85 ± 0.87	0.255
Framingham risk score (%)				0.0001
<6	119 (25.9%)	8 (7.5%)	111 (31.4%)	
≧6	340 (74.1%)	98 (92.5%)	242 (68.6%)	
Follow-up period (years)	4.67 ± 2.46	5.71 ± 2.68	4.35 ± 2.31	0.0001
CAC score in the final round (median, range)	6.24 (0,0–431)	27.29 (13,1–431)	0 (0,0)	0.0001

*BMI* body mass index, *CAC* coronary artery calcification, *HDL-C* high density lipoprotein cholesterol, *LDL-C* low density lipoprotein cholesterol, *HbA1c* Glycosylated hemoglobin A1c

### Frequency and temporal change of CAC progression from zero to > 0

Of the 459 included participants with a baseline CAC score of zero, CAC progression was observed in 106 (23.09%) participants during the follow-up period of 5.71 ± 2.68 years. Male gender, higher Framingham risk score rank according to 10, 20 and 30%, higher age rank (age cutoff of 40, 50 and 65 years) at the baseline were significantly associated with the risk of CAC progression to > 0. The cumulative proportion of CAC progression in this study cohort stratified by gender, Framingham risk score rank and age rank is summarized in Table 2. The 5-year progression rate of male group subjects was 28.29%, which was significantly higher than female group subjects (12.16%). For CAC progression, the mean progression time for male was 7.93 ± 0.27 years and the median progression was 7.86 ± 0.47 years; The female group had a mean progression time of 9.70 ± 0.40 years and a median of 10.56 ± 0.45 years. There was a significant difference in progression periods between the two groups (log-rank test *p* < 0.0001). In term of Framingham risk score rank, the higher Framingham risk score group had shorter conversion time of CAC progression than the lower Framingham risk score group (Framingham risk score cut-off values of 10, 20, and 30%, log-rank test *p* = 0.0001, 0.0001, 0.007). In term of age rank, the higher age rank group had shorter conversion time of CAC progression than the lower age rank group (age rank cutoff values of 40, 50, and 65 yrs. log-rank test *p* = 0.044, 0.025, 0.006).

**Table 2:** CAC conversion rates for subjects with a baseline CAC score of zero stratified according to traditional cardiovascular risk factors

Risk Factor	Converted	*P* value	Time to Conversion, yrs. (mean)	Time to Conversion, yrs. (median)	*P* value
Female	18/148 (12.16%)	0.0001	9.70 ± 0.40	10.56 ± 0.45	0.0001
Male	88/311 (28.29%)		7.93 ± 0.27	7.86 ± 0.47	
Framingham risk score (%)					
<10	23/194 (11.85%)	0.001	9.98 ± 0.36	10.96 ± 0.50	0.0001
≧10	83/265 (31.32%)		7.48 ± 0.26	7.49 ± 0.62	
<20	68/349 (19.48%)	0.002	9.00 ± 0.27	10.29 ± 0.73	0.0001
≧20	38/110 (34.54%)		6.85 ± 0.34	6.92 ± 0.65	
<30	85/400 (21.25%)	0.02	8.68 ± 0.25	9.72 ± 0.94	0.007
≧30	21/59 (35.59%)		7.11 ± 0.47	7.46 ± 1.05	
Age, yrs					
<40	4/35 (11.42%)	0.098	10.24 ± 0.90	–	0.044
≧40	102/424 (24.05%)		8.27 ± 0.23	8.52 ± 0.62	
<50	35/194 (18.04%)	0.033	9.07 ± 0.39	9.72 ± 1.02	0.025
≧50	71/265 (26.79%)		80.2 ± 0.27	8.48 ± 0.62	
<65	93/425 (21.88%)	0.035	8.55 ± 0.24	9.03 ± 0.75	0.006
≧65	13/34 (38.23%)		6.84 ± 0.81	5.94 ± 0.97	

*CAC* coronary artery calcification

### Cox regression for predictors of CAC progression

**Fig. 2:**
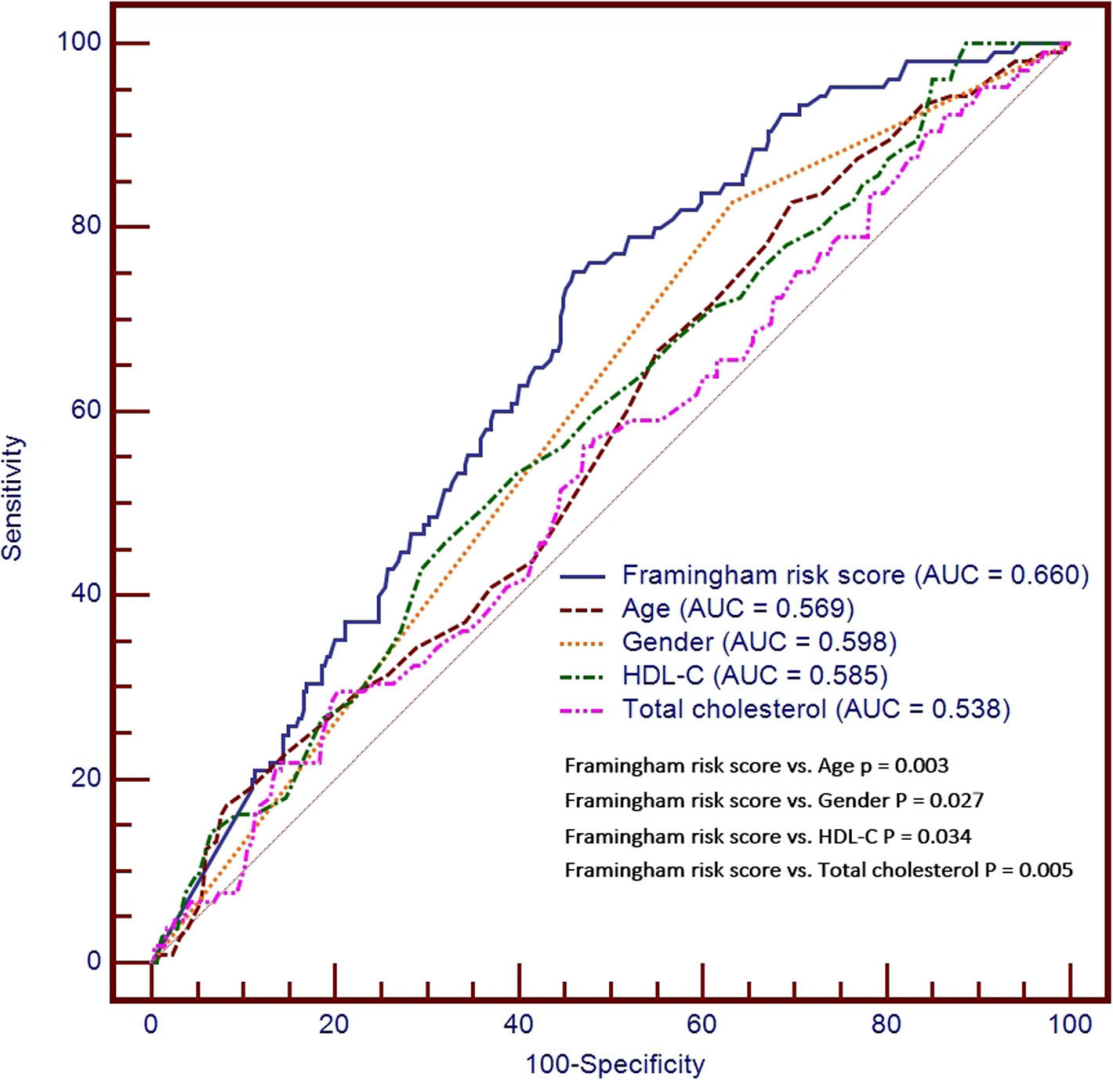
Receiver operating characteristic analysis to compare the diagnostic performance of Framingham risk score, age, male gender, HDL-C, and total cholesterol for prediction of CAC progression. Among these parameters, Framingham risk score with the cut-off ≥11.1 had the better discriminative ability to predict CAC progression compared to the other parameters with a sensitivity of 75.24% and specificity of 53.95% (AUC =0.660, PPV = 32.60%; NPV = 88.00%)

Among subjects with a zero CAC score at baseline, model 1 showed that age, male gender, HDL-C, and total cholesterol were significantly associated with CAC progression. For Framingham risk score stratification, model 2 showed that Framingham risk score were significantly associated with CAC progression shown in Table 3. Among these parameters, Framingham risk score had the better discriminative ability to predict CAC progression with a sensitivity of 75.24% and specificity of 53.95% (PPV = 32.60%; NPV = 88.00%) shown in Supplement Table 1. Framingham risk score had the better discriminative ability to predict CAC progression compared to the other biochemical markers. We further used the Youden index test and found an optimum cut-off value for Framingham risk score of ≥ 11.1 with the highest discriminant ability than other values (AUC = 0.660) shown in Fig. 2. Among these potential predictive parameters, age and male gender were the two most sensitive signs. However, the HDL-C and total cholesterol were the two most specific signs.

**Table 3:** Multivariate Cox regression for CAC progression in the study cohort with a baseline CAC score of zero

	Model 1				Model 2		
Variable	HR	95% CI	P	Variable	HR	95% CI	P
Age	1.057	1.030–1.085	< 0.0001	Framingham risk score (%)	1.056	1.035–1.078	< 0.0001
Gender (male)	2.284	1.273–4.099	0.006				
LDL-C (mg/dL)	0.990	0.977–1.003	0.147				
HDL-C (mg/dL)	0.976	0.953–0.999	0.043				
Total cholesterol (mg/dL)	1.016	1.004–1.028	0.008				
Triglyceride (mg/dL)	0.998	0.995–1.002	0.362				
Diabetes	1.092	0.621–1.922	0.759				
Hypertension	1.211	0.793–1.847	0.376				
Current smoking	1.258	0.791–2.000	0.332				

*CAC* coronary artery calcification, *CI* confidence interval, *HDL-C* high density lipoprotein cholesterol, *LDL-C* low density lipoprotein cholesterol, *HR* Hazard Ratio

### Cox regression for predictors of double zero score with obstructive coronary artery atherosclerosis

**Fig. 3:**
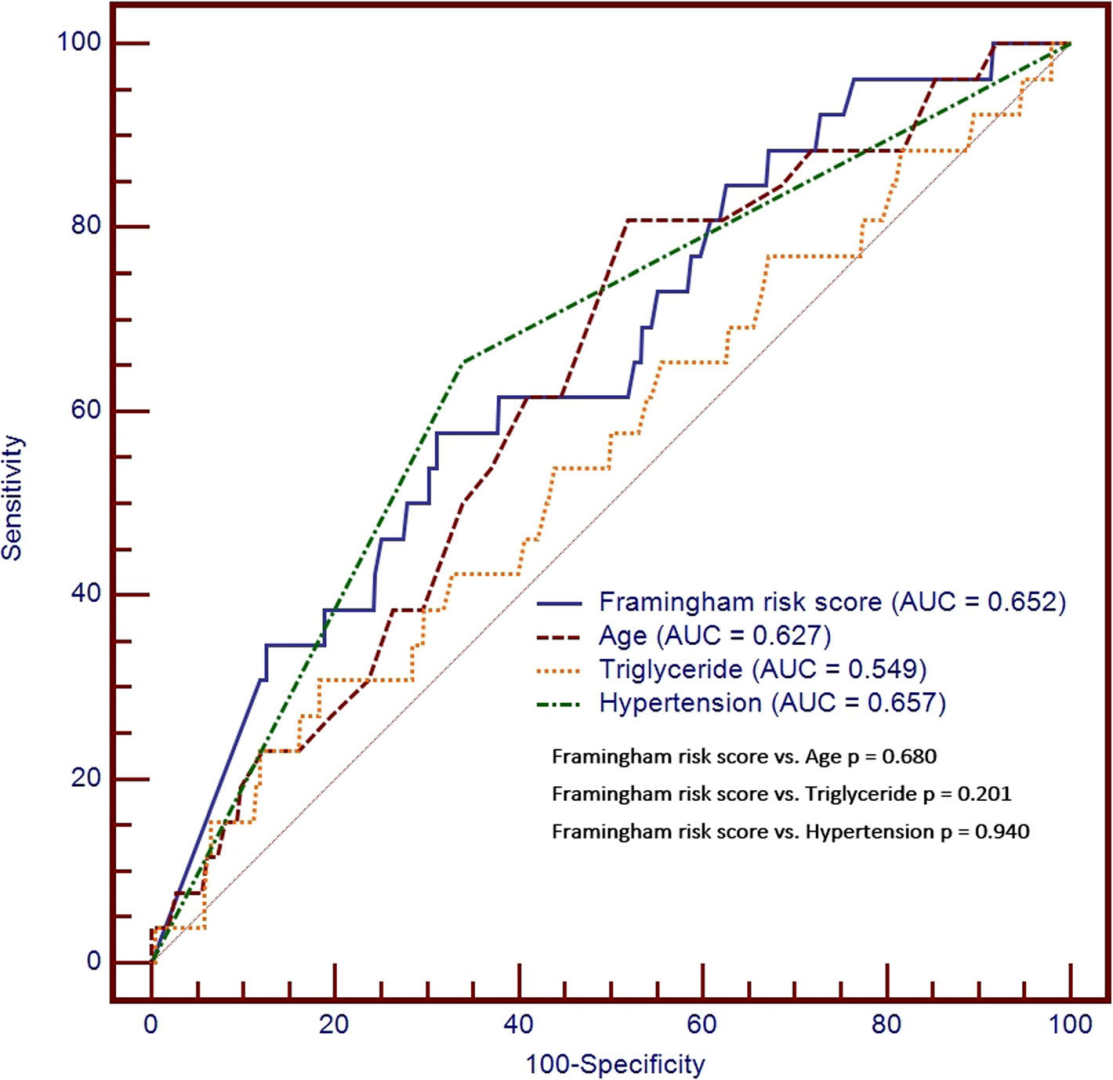
Receiver operating characteristic analysis to compare the diagnostic performance of Framingham risk score, age, triglyceride and hypertension for prediction of double zero score with obstructive coronary artery atherosclerosis. Among these parameters, there was no significant difference in diagnostic performance for double zero score with obstructive coronary artery atherosclerosis prediction

Of the 353 subjects with the double zero CAC score in this cohort, there were 26 subjects with obstructive coronary artery atherosclerosis events diagnosed by coronary CT angiography in the final round of the CT exam. Model 1 showed that older age, triglyceride, and hypertension were significantly associated with these events of double zero score with obstructive coronary artery atherosclerosis. For Framingham risk score stratification, model 2 showed that Framingham risk score were significantly associated with these events (double zero score with obstructive coronary artery atherosclerosis) shown in Table 4. We further used the Youden index test and found an optimum cut-off value for Framingham risk score < 16.9 with the high negative predictive value (NPV =96.4%) shown in Supplement Table 2 and Fig. 3 (AUC = 0.652; sensitivity =57.69%; specificity =68.82%).

**Table 4:** Multivariate Cox regression for obstructive coronary artery atherosclerosis in the study cohort with double zero score

	Model 1				Model 2		
Variable	HR	95% CI	P	Variable	HR	95% CI	P
Age	1.065	1.013–1.119	0.014	Framingham risk score (%)	1.055	1.014–1.098	0.008
Gender (male)	1.150	0.398–3.323	0.796				
LDL-C (mg/dL)	1.025	0.984–1.067	0.240				
HDL-C (mg/dL)	1.026	0.979–1.075	0.283				
Total cholesterol (mg/dL)	0.982	0.947–1.017	0.303				
Triglyceride (mg/dL)	1.005	1.001–1.009	0.025				
Diabetes	1.746	0.699–4.363	0.233				
Hypertension	2.482	1.047–5.884	0.039				
Current smoking	0.97	0.374–2.520	0.951				

*CAC* coronary artery calcification, *CI* confidence interval, *LDL-C* low density lipoprotein cholesterol, *HDL-C* high density lipoprotein cholesterol, *HR* Hazard Ratio

## Discussion

This is a retrospective study for the first time investigating the natural course of coronary atherosclerosis burden in asymptomatic Asian population with an initial CAC score of zero in term of CAC progression and double zero score with obstructive coronary artery atherosclerosis. In this study, we demonstrated five major findings. The first one is that CAC progression rate was about 23.09% in low to intermediate risk Asian population with baseline zero score during the follow-up period of 5.71 ± 2.68 years. Second, older age, male gender, HDL-C, total cholesterol and higher Framingham risk score were independently associated with CAC progression and shorter acceleration time of CAC progression. Third, for CAC progression, Framingham risk score had the better diagnostic ability performance with an AUC of 0.660 (sensitivity of 75.24%; specificity of 53.95%) than other parameters. Forth, for double zero score with obstructive coronary artery atherosclerosis event, older age, triglyceride, hypertension and Framingham risk score were independent important risk factors for event prediction. Finally, for rule out double zero score with obstructive coronary artery atherosclerosis event, Framingham risk score with an optimum cut-off value of less than 16.9 may be a relatively acceptable parameter with high negative predictive (NPV = 96.4%) value for rule out obstructive coronary artery atherosclerosis scenario (sensitivity of 57.69%; specificity of 68.82%).

The accumulating evidences from previous studies regarding CAC progression in the general population suggest that zero CAC score at the baseline scan provides the 5-year warranty period for asymptomatic subjects, especially in the Western population [9, 14–16]. However, there is limited research regarding the CAC progression in the Asian population with a baseline zero CAC score [8, 17]. A previous study demonstrated that a baseline CAC score of zero is associated with the low probability of CAC progression in Korean population with a nonlinear increasing trend over time [17]. Our results are in line with previous studies. Thus, the beneficial prognosis of zero CAC score for the warranty period of 5 years to prevent CAC progression is also further supported in the Asian population in our study result. In addition, our study demonstrated that age, male gender, HDL-C, total cholesterol and Framingham risk score are important predictors of CAC progression. However, Lee et al. previously reported that age, male sex, waist circumference, diabetes, and low-density lipoprotein cholesterol level independently associated with an increased risk of annualized CAC progression in Korean population [17]. In addition, age > 40 years, diabetes, and smoking are independently associated with the risk of conversion to a CAC score > 0 in Western population with a baseline CAC score of zero in the previous study by Min et al. [16]. Therefore, the difference between these studies could be partly explained by ethnic differences and study endpoints. However, the present study, which focuses exclusively on the Chinese population with a baseline zero CAC score, provides important information regarding Framingham risk score ≥ 11.1 associated with a high risk of CAC progression in the Chinese population in the warranty period. Cardiovascular risk assessment by Framingham risk score stratification could help guide the management and prevent subclinical coronary atherosclerosis.

Lehmann et al. previously reported that double-zero CAC scans in a 5-year period mean an excellent prognosis for cardiovascular events [9]. However, recent studies have showed that absence of coronary artery calcification does not completely exclude obstructive coronary artery disease, but the prevalence is variable between 1 to 20% [18–23]. Our study results demonstrated that older age, triglyceride, hypertension and Framingham risk score were the independent most important risk factors for prediction of double-zero CAC with obstructive coronary artery atherosclerosis. For prediction of double-zero CAC with coronary artery atherosclerosis, the Framingham risk score may be a relatively acceptable parameter at an optimum cut-off value of less than 16.9 for rule out double-zero CAC with obstructive coronary artery atherosclerosis. However, Framingham risk score was a just relatively acceptable parameter among these parameters for events prediction because of high NPV of 96.4% in the clinical setting of low to intermediate cardiovascular risk. In this study we have demonstrated that Framingham risk score significantly associated with CAC progression or double zero score with obstructive coronary artery atherosclerosis. But the poor discrimination ability may be revealed by the AUC range of 0.6 to 0.7 in the prediction of CAC progression or double zero score with obstructive coronary artery atherosclerosis. These questions raised by this study warrant further investigation of a better risk score model.

Currently, there is controversy about the prognosis of obstructive CAD in subjects with a zero CAC score [18–20]. Previous studies demonstrated that zero CAC score can’t be used to totally exclude obstructive CAD or adverse cardiac events in symptomatic Korean subjects [20]. However Mittal et al. previously showed that the presence of non-calcified atheroma/plaques on coronary CT angiography in subjects with zero CAC score did not affect the prognostic outcome [19]. Therefore, our proposed “Framingham risk score threshold less than 16.9” may help confirm subjects at low risk of double zero score with obstructive coronary artery atherosclerosis to improve cardiovascular risk stratification and reclassification. Previous studies also demonstrated that 1.9 ~ 4.3% subjects found to have obstructive CAD ≧50% among symptomatic subjects with zero CAC score [20, 27, 28]. This study supported previous findings and could further help to reclassify high-risk group with high Framingham risk score. Therefore, this high-risk group with zero CAC score could benefit prognostically from optimal medical treatment as demonstrated in the Scottish Computed Tomography of the HEART Trial (SCOTHEART) [29, 30].

There are some limitations in this study. First, this is a retrospective study based in a single-center with self-referral healthy population of low to intermediate cardiovascular risk. Therefore, the risk of selection bias should be considered.

Second, we did not specifically investigate the clinical outcomes or mortality for the primary outcome analysis. Therefore, future studies are warranted to investigate these issues based on multicenter prospective studies to determine the prognostic outcome of CAC progression and double zero score with obstructive coronary artery atherosclerosis in Asian population. Third, a potential problem of overfitting the model should be concerned because of a small number of events for double zero score with obstructive coronary artery atherosclerosis. Therefore, large prospective cohort studies are needed to validate this study outcome.

## Conclusion

The present study results demonstrate that the Asian population in Taiwan with low to intermediate risk has a low conversion rate of CAC progression within the 5-year warranty period. Framingham risk score ≥ 11.1 associated with a high risk of CAC progression in the warranty period. In addition, a zero CAC score may serve as a gatekeeper for cardiovascular event prevention. For CAC progression prediction, Framingham risk score with the cutoff < 11.1 may help confirm subjects at low risk to improve cardiovascular risk stratification and reclassification in the field of preventive cardiology.

## Supplementary information

**Additional file 1: Supplement Table 1.** The diagnostic performance among CAD risk parameters used for predicting CAC progression in the study cohort with a baseline CAC score of zero. **Supplement Table 2.** The diagnostic performance among CAD risk parameters used for predicting in the study cohort with double zero score with obstructive coronary artery atherosclerosis.

## Data Availability

The datasets generated and/or analyzed during the current study are not publicly available because the information and data of the study population were extracted from Hospital Information System and were recorded manually in EXCEL to form our private database. But the data are available from the corresponding author on reasonable request.

## Abbreviations

CAC: Coronary artery calcium
CAD: Coronary artery disease
BMI: Body mass index
HDL-C: High-density lipoprotein cholesterol
LDC-C: Low*-*density lipoprotein cholesterol
HbA1c: Hemoglobin A1c
SCCT: Society of cardiovascular computed tomography
HU: Hounsfield units
VIFs: Variance inflation factors
ECG: Electrocardiogram
positive LR: Positive likelihood ratio
negative LR: Negative likelihood ratio
PPV: Positive predictive value
NPV: Negative predictive value
SD: Standard deviation

## References

[1] Engbers EM, Timmer JR, Ottervanger JP (2017). Coronary artery calcium score as a gatekeeper in the non-invasive evaluation of suspected coronary artery disease in symptomatic patients. J Nuclear Cardiol.

[2] Torres FS, Venkatesh V, Nguyen ET, Jimenez-Juan L, Crean AM. Coronary Calcium Scan Acquisition Before Coronary CT Angiography: Limited Benefit or Useful Addition? American Journal of Roentgenology. 2013;200(1):66–73. 10.2214/AJR.12.8643 23255743

[3] Palumbo AA, Maffei E, Martini C, Tarantini G, Di Tanna GL, Berti E, Grilli R, Casolo G, Brambilla V, Cerrato M (2009). Coronary calcium score as gatekeeper for 64-slice computed tomography coronary angiography in patients with chest pain: per-segment and per-patient analysis. Eur Radiol.

[4] Youssef G, Budoff MJ (2012). Coronary artery calcium scoring, what is answered and what questions remain. Cardiovasc Diagn Ther.

[5] Berry JD, Liu K, Folsom AR, Lewis CE, Carr JJ, Polak JF, Shea S, Sidney S, O'Leary DH, Chan C (2009). Prevalence and progression of subclinical atherosclerosis in younger adults with low short-term but high lifetime estimated risk for cardiovascular disease: the coronary artery risk development in young adults study and multi-ethnic study of atherosclerosis. Circulation.

[6] Budoff MJ, Hokanson JE, Nasir K, Shaw LJ, Kinney GL, Chow D, Demoss D, Nuguri V, Nabavi V, Ratakonda R (2010). Progression of coronary artery calcium predicts all-cause mortality. J Am Coll Cardiol Img.

[7] Budoff MJ, McClelland RL, Nasir K, Greenland P, Kronmal RA, Kondos GT, Shea S, Lima JAC, Blumenthal RS (2009). Cardiovascular events with absent or minimal coronary calcification: the multi-ethnic study of atherosclerosis (MESA). Am Heart J.

[8] Lee JH, Han D, OH B, Rizvi A, Gransar H, Park HB, Park HE, Choi SY, Chun EJ, Sung J (2016). Warranty period of zero Coronary artery calcium score for predicting all-cause mortality according to cardiac risk burden in asymptomatic Korean adults. Circulation J.

[9] Lehmann N, Erbel R, Mahabadi AA, Rauwolf M, Mohlenkamp S, Moebus S, Kalsch H, Budde T, Schmermund A, Stang A (2018). Value of progression of Coronary artery calcification for risk prediction of Coronary and cardiovascular events: result of the HNR study (Heinz Nixdorf recall). Circulation.

[10] Valenti V, Ó Hartaigh B, Heo R, Cho I, Schulman-Marcus J, Gransar H, Truong QA, Shaw LJ, Knapper J, Kelkar AA et al: A 15-year warranty period for asymptomatic individuals without Coronary artery calcium: a prospective follow-up of 9,715 individuals. J Am Coll Cardiol Img 2015;8(8):900–909. 10.1016/j.jcmg.2015.01.025 PMC4537357 26189116

[11] Arnett DK, Blumenthal RS, Albert MA, Buroker AB, Goldberger ZD, Hahn EJ, Himmelfarb CD, Khera A, Lloyd-Jones D, McEvoy JW, et al. 2019 ACC/AHA Guideline on the Primary Prevention of Cardiovascular Disease: A Report of the American College of Cardiology/American Heart Association Task Force on Clinical Practice Guidelines. Circulation. 2019;140(11):e596–e646. 10.1161/CIR.0000000000000678 PMC7734661 30879355

[12] Wu F-Z, Wu CC, Kuo P-L, Wu M-T (2016). Differential impacts of cardiac and abdominal ectopic fat deposits on cardiometabolic risk stratification. BMC Cardiovasc Disord.

[13] Wu F-Z, Chou K-J, Huang Y-L, Wu M-T (2014). The relation of location-specific epicardial adipose tissue thickness and obstructive coronary artery disease: systemic review and meta-analysis of observational studies. BMC Cardiovasc Disord.

[14] Gopal A, Nasir K, Liu ST, Flores FR, Chen L, Budoff MJ (2007). Coronary calcium progression rates with a zero initial score by electron beam tomography. Int J Cardiol.

[15] Koulaouzidis G, Charisopoulou D, Maffrett S, Tighe M, Jenkins PJ, McArthur T (2013). Coronary artery calcification progression in asymptomatic individuals with initial score of zero. Angiology.

[16] Min JK, Lin FY, Gidseg DS, Weinsaft JW, Berman DS, Shaw LJ, Rozanski A, Callister TQ (2010). Determinants of coronary calcium conversion among patients with a normal coronary calcium scan: what is the "warranty period" for remaining normal?. J Am Coll Cardiol.

[17] Lee W, Yoon YE, Kwon O, Lee H, Park HE, Chun EJ, Choi SY, Cho GY, Chang HJ (2019). Evaluation of Coronary artery calcium progression in asymptomatic individuals with an initial score of zero. Korean Circ J.

[18] de Carvalho MS, de Araujo GP, Garcia-Garcia HM, de Sousa PJ, Dores H, Ferreira A, Cardim N, Carmo MM, Aleixo A, Mendes M (2013). Prevalence and predictors of coronary artery disease in patients with a calcium score of zero. Int J Cardiovasc Imaging.

[19] Mittal TK, Pottle A, Nicol E, Barbir M, Ariff B, Mirsadraee S, Dubowitz M, Gorog DA, Clifford P, Firoozan S (2017). Prevalence of obstructive coronary artery disease and prognosis in patients with stable symptoms and a zero-coronary calcium score. Eur Heart J Cardiovasc Imaging.

[20] Kim YJ, Hur J, Lee H-J, Chang H-J, Nam JE, Hong YJ, Kim HY, Lee JW, Choi BW (2012). Meaning of zero coronary calcium score in symptomatic patients referred for coronary computed tomographic angiography. Eur Heart J Cardiovasc Imaging.

[21] Sarwar A, Shaw LJ, Shapiro MD, Blankstein R, Hoffmann U, Cury RC, Abbara S, Brady TJ, Budoff MJ, Blumenthal RS (2009). Diagnostic and prognostic value of absence of coronary artery calcification. J Am Coll Cardiol Img.

[22] Rubinshtein R, Gaspar T, Halon DA, Goldstein J, Peled N, Lewis BS (2007). Prevalence and extent of obstructive coronary artery disease in patients with zero or low calcium score undergoing 64-slice cardiac multidetector computed tomography for evaluation of a chest pain syndrome. Am J Cardiol.

[23] Gottlieb I, Miller JM, Arbab-Zadeh A, Dewey M, Clouse ME, Sara L, Niinuma H, Bush DE, Paul N, Vavere AL (2010). The absence of coronary calcification does not exclude obstructive coronary artery disease or the need for revascularization in patients referred for conventional coronary angiography. J Am Coll Cardiol.

[24] Agatston AS, Janowitz WR, Hildner FJ, Zusmer NR, Viamonte M, Detrano R (1990). Quantification of coronary artery calcium using ultrafast computed tomography. J Am Coll Cardiol.

[25] Abbara S, Blanke P, Maroules CD, Cheezum M, Choi AD, Han BK, Marwan M, Naoum C, Norgaard BL, Rubinshtein R (2016). SCCT guidelines for the performance and acquisition of coronary computed tomographic angiography: a report of the society of cardiovascular computed tomography guidelines committee: endorsed by the north American Society for Cardiovascular Imaging (NASCI). J Cardiovasc Comput Tomography.

[26] Cury RC, Abbara S, Achenbach S, Agatston A, Berman DS, Budoff MJ, Dill KE, Jacobs JE, Maroules CD, Rubin GD (2016). CAD-RADS (TM) Coronary artery disease - reporting and data system. An expert consensus document of the Society of Cardiovascular Computed Tomography (SCCT), the American College of Radiology (ACR) and the north American Society for Cardiovascular Imaging (NASCI). Endorsed by the American College of Cardiology. J Cardiovasc Computed Tomography.

[27] Villines TC, Hulten EA, Shaw LJ, Goyal M, Dunning A, Achenbach S, Al-Mallah M, Berman DS, Budoff MJ, Cademartiri F (2011). Prevalence and severity of coronary artery disease and adverse events among symptomatic patients with coronary artery calcification scores of zero undergoing coronary computed tomography angiography: results from the CONFIRM (Coronary CT Angiography Evaluation for Clinical Outcomes: An International Multicenter) registry. J Am College Cardiol.

[28] Wang X, Le EPV, Rajani NK, Hudson-Peacock NJ, Pavey H, Tarkin JM, Babar J, Williams MC, Gopalan D, Rudd JHF (2019). A zero coronary artery calcium score in patients with stable chest pain is associated with a good prognosis, despite risk of non-calcified plaques. Open Heart.

[29] Adamson PD, Hunter A, Williams MC, Shah ASV, McAllister DA, Pawade TA, Dweck MR, Mills NL, Berry C, Boon NA (2018). Diagnostic and prognostic benefits of computed tomography coronary angiography using the 2016 National Institute for health and care excellence guidance within a randomised trial. Heart (British Cardiac Society).

[30] Coronary CT (2018). Angiography and 5-year risk of myocardial infarction. N Engl J Med.

