# Supplementary material for: Natural course of coronary artery calcium progression in Asian population with an initial score of zero
Source: BMC Cardiovasc Disord. 2020 May 6;20:212. doi: 10.1186/s12872-020-01498-x (PMC7204036; doi:10.1186/s12872-020-01498-x)
Supplement: Supplementary file 1 — Additional file 1: Supplement Table 1. The diagnostic performance among CAD risk parameters used for predicting CAC progression in the study cohort with a baseline CAC score of zero. Supplement Table 2. The diagnostic performance among CAD risk parameters used for predicting in the study cohort with double zero score with obstructive coronary artery atherosclerosis. [file 12872_2020_1498_MOESM1_ESM.docx]

**Title**

Natural course of coronary artery calcium progression in Asian population with an initial score of zero

**Authors names:**

Yi-Wen Shen^1,2,+^, Yun-Ju Wu^1,+^, Yi-Chi Hung^1,3,+^, Chia-Chi Hsiao^1,3+^,Shan-Ho Chan^3^, Guang-Yuan Mar^4^, Ming-Ting Wu^1,5^, Fu-Zong Wu^1,3,5,*^

**Department and institutions**

^1^Department of Radiology, Kaohsiung Veterans General Hospital, Kaohsiung, Taiwan.

^2^Department of Medical Imaging and Radiological Sciences, Kaohsiung Medical University, Kaohsiung, Taiwan.

^3^Department of Medical Imaging and Radiology, Shu-Zen Junior College of Medicine and Management, Kaohsiung, Taiwan.

^4^Physical Examination Center, Kaohsiung Veterans General Hospital, Kaohsiung, Taiwan.

^5^Faculty of Medicine, School of Medicine, National Yang-Ming University, Taipei, Taiwan.

**Contact info:**

^*^Fu-Zong, Wu, MD

Department of Radiology, Kaohsiung Veterans General Hospital, Kaohsiung, Taiwan. 386 Ta-Chung 1st Road, 813 Kaohsiung, Taiwan; Faculty of Medicine, School of Medicine, National Yang-Ming University, Taipei, Taiwan; Department of Medical Imaging and Radiology, Shu-Zen Junior College of Medicine and Management, Kaohsiung, Taiwan. Email: cmvwu1029@gmail.com

| Supplement Table 1. The diagnostic performance among CAD risk parameters used for predicting CAC progression in the study cohort with a baseline CAC score of zero | | | | | | | | | |
| --- | --- | --- | --- | --- | --- | --- | --- | --- | --- |
| Variables | ROC curve parameters | | | | | | | | |
|  | Cut-off | AUC | 95% CI | Sensitivity | Specificity | PPV (%) | NPV (%) | LR+ | LR- |
| Age | ≧46 | 0.569 | 0.522-0.614 | 82.86 | 30.23 | 26 | 85.6 | 1.19 | 0.57 |
| Gender (male) | (male) | 0.598 | 0.551-0.643 | 82.86 | 36.72 | 28 | 87.8 | 1.31 | 0.47 |
| Framingham risk score (%) | ≧11.1 | 0.66 | 0.614-0.703 | 75.24 | 53.95 | 32.6 | 88 | 1.63 | 0.46 |
| HDL-C (mg/dL) | ≦40 | 0.585 | 0.538-0.631 | 45.71 | 68.08 | 29.8 | 80.9 | 1.43 | 0.8 |
| Total cholesterol (mg/dL) | ≧234 | 0.538 | 0.491-0.584 | 29.52 | 79.66 | 30.1 | 79.2 | 1.45 | 0.88 |
| CAD, coronary artery disease; CAC, coronary artery calcification; ROC, receiver operating characteristic curve; HDL-C, high density lipoprotein cholesterol; AUC, area under the receiver operating characteristic; PPV, positive predictive value; NPV, negative predictive value; LR+, positive likelihood ratio; LR-, negative likelihood ratio. | | | | | | | | | |

| Supplement Table 2. The diagnostic performance among CAD risk parameters used for predicting in the study cohort with double zero score with obstructive coronary artery atherosclerosis | | | | | | | | | |
| --- | --- | --- | --- | --- | --- | --- | --- | --- | --- |
| Variables | ROC curve parameters | | | | | | | | |
|  | Cut-off | AUC | 95% CI | Sensitivity | Specificity | PPV (%) | NPV (%) | LR+ | LR- |
| Age | ≧50 | 0.627 | 0.581-0.671 | 80.77 | 48.04 | 8.5 | 97.7 | 1.55 | 0.4 |
| Framingham risk score (%) | ≧16.9 | 0.652 | 0.607-0.696 | 57.69 | 68.82 | 10 | 96.4 | 1.85 | 0.61 |
| Triglyceride (mg/dL) | ≧213 | 0.549 | 0.502-0.595 | 30.77 | 81.52 | 9.1 | 95.1 | 1.67 | 0.85 |
| Hypertension | (+) | 0.657 | 0.612-0.701 | 65.38 | 66.05 | 10.4 | 96.9 | 1.93 | 0.52 |
| CAD, coronary artery disease; ROC, receiver operating characteristic curve; AUC, area under the receiver operating characteristic; PPV, positive predictive value; NPV, negative predictive value; LR+, positive likelihood ratio; LR-, negative likelihood ratio. | | | | | | | | | |
